# Identification of the genetic determinants of *Salmonella enterica *serotype Typhimurium that may regulate the expression of the type 1 fimbriae in response to solid agar and static broth culture conditions

**DOI:** 10.1186/1471-2180-8-126

**Published:** 2008-07-25

**Authors:** Yin-Ching Chuang, Ke-Chuan Wang, Yi-Tseng Chen, Chia-Huei Yang, Shang-Chin Men, Chia-Chun Fan, Li-Huan Chang, Kuang-Sheng Yeh

**Affiliations:** 1 Department of Medical Research, Chi Mei Medical Center, 901 Chung Hwa Road, Yong Kang City, Tainan 710, Taiwan; 2 Graduate Institute of Medical Sciences, College of Medicine, Taipei Medical University, 250 Wu-Hsing Street, Taipei 110, Taiwan; 3 Department of Microbiology and Immunology, School of Medicine, College of Medicine, Taipei Medical University, 250 Wu-Hsing Street, Taipei 110, Taiwan

## Abstract

**Background:**

Type 1 fimbriae are the most commonly found fimbrial appendages on the outer membrane of *Salmonella enterica *serotype Typhimurium. Previous investigations indicate that static broth culture favours *S*. Typhimurium to produce type 1 fimbriae, while non-fimbriate bacteria are obtained by growth on solid agar media. The phenotypic expression of type 1 fimbriae in *S*. Typhimurium is the result of the interaction and cooperation of several genes in the *fim *gene cluster. Other gene products that may also participate in the regulation of type 1 fimbrial expression remain uncharacterized.

**Results:**

In the present study, transposon insertion mutagenesis was performed on *S*. Typhimurium to generate a library to screen for those mutants that would exhibit different type 1 fimbrial phenotypes than the parental strain. Eight-two mutants were obtained from 7,239 clones screened using the yeast agglutination test. Forty-four mutants produced type 1 fimbriae on both solid agar and static broth media, while none of the other 38 mutants formed type 1 fimbriae in either culture condition. The flanking sequences of the transposons from 54 mutants were cloned and sequenced. These mutants can be classified according to the functions or putative functions of the open reading frames disrupted by the transposon. Our current results indicate that the genetic determinants such as those involved in the fimbrial biogenesis and regulation, global regulators, transporter proteins, prophage-derived proteins, and enzymes of different functions, to name a few, may play a role in the regulation of type 1 fimbrial expression in response to solid agar and static broth culture conditions. A complementation test revealed that transforming a recombinant plasmid possessing the coding sequence of a NAD(P)H-flavin reductase gene *ubiB *restored an *ubiB *mutant to exhibit the type 1 fimbrial phenotype as its parental strain.

**Conclusion:**

Genetic determinants other than the *fim *genes may involve in the regulation of type 1 fimbrial expression in *S*. Typhimurium. How each gene product may influence type 1 fimbrial expression is an interesting research topic which warrants further investigation.

## Background

Salmonellosis is one of the important causes of food-borne diseases throughout the world [1]. *Salmonella enterica *contains more than 2,300 serotypes among which Typhimurium is an important causative agent of gastroenteritis. Adhesion of bacteria to the host epithelial cells is a prerequisite step in establishing infection. Specific adhesion requires the interaction of specialized complementary molecules in a ligand-receptor interaction between bacterial surfaces and host tissues [2]. Proteinaceous hair-like structures called fimbriae on the surface of bacteria have been implicated in such an event [3]. Many members of the family *Enterobacteriaceae *including *Salmonella *produce type 1 fimbriae, the most commonly found type of fimbrial appendages [4]. Type 1 fimbriae comprise a family of rod-shaped organelles which are 7 nm in diameter and 0.2–2.0 μm long [5]. Type 1 fimbriae adhere to different cell types including erythrocytes, leukocytes, intestinal cells, respiratory cells, protozoa, yeast, fungal hyphae, and plant root hairs [6]. Several studies indicated that type 1 fimbriae also contribute to virulence [7-9]. For example, type 1 fimbriae expressing *S*. Typhimurium caused persistent infection in swine [7]. Type 1 fimbriae may also modulate the bacterial tropism to the gut of the host [8], and type 1-fimbriated *Salmonella *were more virulent than the *fim*-minus ones [9]. The fact that more than 80% of *Salmonella *isolates produce this fimbrial type suggests that type 1 fimbriae play an important role at some stage in the life cycle of bacteria [10].

Phenotypic variation of the expression of type 1 fimbriae in *S*. Typhimurium was first characterized by Old et al. [11,12]. They described a biphasic growth pattern associated with the outgrowth of fimbriate bacteria incubated in static, liquid broth culture. Briefly, strongly type 1 fimbriate phase *S*. Typhimurium cells were isolated following serial passage every 48 h in static broth culture. Non-fimbriate phase bacteria were obtained by growth on solid media.

Current data indicate that the phenotypic expression of type 1 fimbriae in *S*. Typhimurium is the result of the interaction and cooperation of several genes in the *fim *gene cluster [13-18]. The FimZ transcriptional factor activates the *fimA *expression by binding to the *fimA *promoter, and in addition, FimZ also positively regulates its own transcription [13]. FimZ requires another co-activator FimY to activate *fimA *expression, however, no evidence has revealed any interaction of FimY and the *fimA *promoter [19]. Interestingly, *fimY *gene possesses five rarely used arginine codon which is recognized by an arginine tRNA gene *fimU*. A *S*. Typhimurium *fimU *mutant did not produce type 1 fimbriae and the *fimY *translation was inhibited [16,17]. *fimU *modulates the *fimY *expression in the translational level. A *fimW *deleted strain, on the contrary, overproduced type 1 fimbriae as compared to the parental strain. This negative regulation was proposed to be mediated through an inhibitory effect on the FimZ protein by a FimW-FimZ protein-protein interaction [15]. Other than gene products within the *fim *gene cluster, McFarland et al. recently reported that a knockout mutation in *lrp*, encoding the leucine-responsive regulatory protein (LRP), was non-fimbriated [20]. They demonstrated that LRP positively regulated type 1 fimbrial expression by binding to the promoter region of *fimZ *[20]. Other gene products that may play a role in type 1 fimbrial expression were seldom characterized. Our long-term goal in the laboratory is focused on finding any genetic determinants, other than those previously described within the *fim *gene cluster, which may also play a role in the phenotypic expression of type 1 fimbriae in *S*. Typhimurium. Initially we were interested in characterizing the genetic elements that may be associated with the ability of *S*. Typhimurium to switch type 1 fimbrial expressions in response to solid agar and static broth culture conditions. We used transposon mutagenesis to generate a library for *S*. Typhimurium and screened mutants that exhibited different type 1 fimbrial phenotypes than the parental strain between solid agar and static broth culture conditions. The flanking DNA fragments of the transposon insertion sites in the mutants of interest were cloned and sequenced. Herein, we report on the genetic determinants in *S*. Typhimurium that may play a role in the regulation of type 1 fimbrial expression between solid agar and static broth culture conditions.

## Results

### Construction of a transposon mutant library of *S*. Typhimurium

In order to identify any genetic determinant that is responsible for *S*. Typhimurium to regulate type 1 fimbrial expression between solid agar and static broth cultures, we used a transposon mutagenesis technique to construct a transposon library. We obtained a library with approximately 8,000 clones.

### Screening of *S*. Typhimurium transposon mutants that exhibited different type 1 fimbrial phenotypes than the parental strain

**Figure 1:**
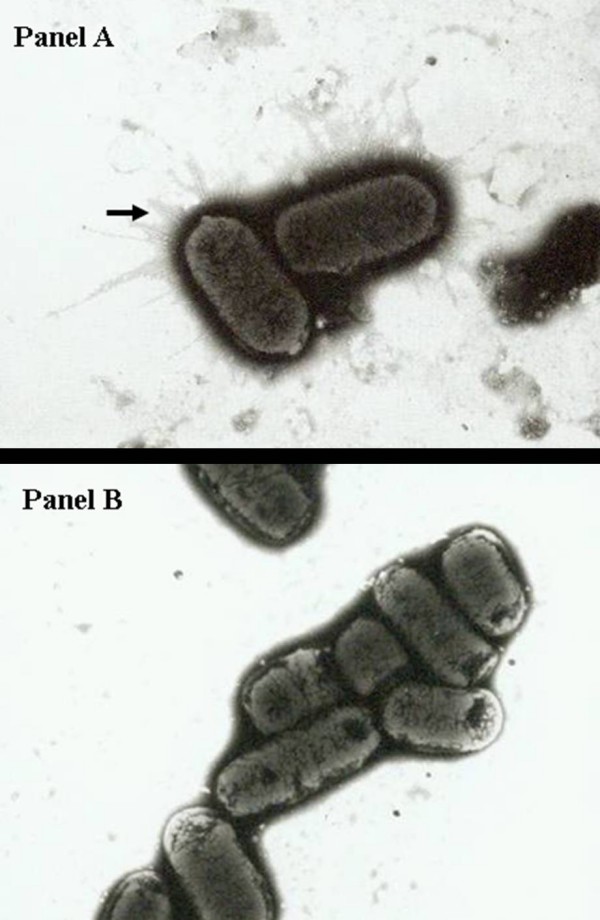
**Observation of type 1 fimbriae in *S*. Typhimurium LB5010 by electron microscopy**. Panel A. *S*. Typhimurium LB5010 cells produced type 1 fimbrial appendages on the outer membrane (arrow) when cultured in LB static broth at 37°C for 48 hr (20,000 ×). Panel B. *S*. Typhimurium LB5010 cells did not produce type 1 fimbrial appendages on the outer membrane when cultured on LB agar at 37°C for 18 hr (20,000 ×). Bacterial cells were negatively stained with 2% of phosphotungstic acid.

**Figure 2:**
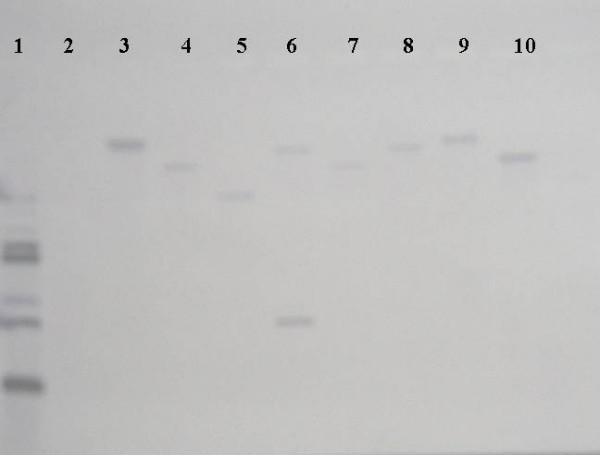
**A representative Southern blot demonstrating hybridization of the genomic DNA cleaved with *Eco*RI with a DIG-labeled 1.3-kb kanamycin-resistance cassette DNA probe**. Lane 1: pUC4K plasmid DNA (positive control); lane 2: *S*. Typhimurium LB5010 (negative control); lanes 3 to 10: K1 to K8 strains, respectively.

In total, 7,239 transposon mutants were screened for those which exhibited different type 1 fimbrial phenotypes as compared to the parental strain *S*. Typhimurium LB5010. The expression of type 1 fimbriae was determined by yeast agglutination test. *S*. Typhimurium LB5010, when grown in static broth culture for 36–48 h, agglutinated *Candida albicans *on a glass slide. These bacterial cells produced type 1 fimbriae on the outer membrane when negatively stained with phosphotungstic acid and observed under electron microscopy (Figure 1, Panel A). On the contrary, *S*. Typhimurium LB5010 did not agglutinate *C. albicans *on a glass slide when bacterial cells were prepared from solid agar medium grown for 16–18 h. The absence of agglutination correlated with the fact that no type 1 fimbriae were observed by electron microscopy (Figure 1, Panel B). Fifty-four mutants verified by Southern blot to have a single unique transposon insertion were selected for further investigation (Figure 2). For those strains with multiple transposon insertions (Figure 2, lane 6 for example) were currently excluded from the present study. This could be due to partial digestion of the genomic DNA or multiple transposons inserted into one bacterial strain. These 54 mutants no longer exhibited the same type 1 fimbrial phenotype as the parental strain, among which 31 mutants produced type 1 fimbriae in both culture conditions, while another 23 mutants produced type 1 fimbriae in neither culture condition. The genes inactivated in the mutants were identified by cloning and sequencing the DNA fragments adjacent to the transposon insertion sites. The DNA sequences were used to search against the genomic sequence of *S*. Typhimurium. The position of the transposon insertion site was determined along with whether or not a transposon insertion site was located in or near an open reading frame (ORF), as predicted by the annotated genomic sequences. In the present study, we only focused on those mutants that demonstrated disruptions in ORFs; these types of mutation are expected to disrupt the functions of the ORFs involved. Table 1 lists those mutants. Transposons which disrupted between ORFs were excluded in the present study.

**Table 1:** *S*. Typhimurium mutants that exhibited different type 1 fimbrial phenotypes than the parental strain

Category	Mutant	Phenotype	Gene^a^	Function
		(agar, broth)		
Fimbrial biosynthesis and regulation	K2	-, -	*fimZ*	Positive regulator of type 1 fimbriae major subunit gene *fimA*
	K14	-, -	*fimY*	Positive regulator of type 1 fimbriae major subunit gene *fimA*
	K40	-, -	*fimA*	Major subunit of type 1 fimbriae
	K66	-, -	*fimD*	Fimbrial usher protein of FimA
	K75	-, -	*fimC*	Fimbrial chaperone protein of FimA
	K44	-, -	*fimH*	Adhesin of type 1 fimbriae
	K70	+, +	*lpfB*	Fimbrial chaperone protein of long polar fimbriae
	K61	+, +	*pefI*	Regulator of plasmid-encoded fimbriae
	K23	+, +	*stbC*	Fimbrial usher protein of Stb fimbriae
Putative cytoplasmic protein	K1	+, +	STM1078	Putative cytoplasmic protein
	K28	+, +	STM4529	Putative cytoplasmic protein
Global regulator	K46	-, -	*lrp*	Leucine-responsive regulatory protein
	K15	-, -	*hupA*	Histone-like DNA binding protein
Membrane associated protein	K17,	+, +	*yhjV*	Putative transporter protein
	K39	-, -	STM1128	Putative sodium/glucose cotransporter
	K45	-, -	*yihO*	Putative glycoside-pentoside-hexuronide (GPH) family transport protein
	K50	+, +	STM2532	Putative inner membrane lipoprotein
	K52	+, +	STM2486	Putative inner membrane protein
	K54	-, -	*yjiH*	Putative inner membrane protein
	K72	+, +	*mgtB*	Mg^2+ ^transporter protein
	K30	+, +	*narK*	Nitrate/nitrite transporter protein
Cell envelope associated protein	K60	+, +	*ytfB*	Putative cell envelope opacity-associated protein A
Periplasmic protein	K69	+, +	*ydbH*	Putative periplasmic protein
Ribosome modulation factor	K56	-, -	*rmf*	Ribosome modulation factor
Type III secretion system	K55	-, -	*ssaB*	Effector protein of type III secretion system
	K59	+, +	*invB*	Chaperone protein for SopE/SopE2
	K63	-, -	*ssaV*	Apparatus protein for intracellular trafficking and secretion
Prophage-derived protein	K11	+, +	STM4200	Putative phage tail protein H
	K33	+, +	*sseI*	Gifsy-2 prophage putative type III secreted effector protein
	K19	-, -	STM1041	Gifsy-2 prophage probable minor tail protein
Sensor protein	K51	+, +	*ssrA*	Sensor component of type III secretion system
	K58	+, +	*yehU*	Sensor component
Transcription termination factor	K73	-, -	*nusA*	Transcription termination factor
Enzymes	K3	-, -	*cafA*	Endoribonuclease
	K5	+, +	*res*	Subunit of type III restriction-modification enzyme
	K8	+, +	STM4467	Putative arginine deiminase
	K16	+, +	STM1627	Alcohol dehydrogenase class III
	K31	+, +	STM2446	Putative iron-dependent peroxidase
	K41	-, -	*nuoE*	NADH dehydrogenase I chain E
	K37	+, +	*yafA*	Putative hydrolase
	K43	-, -	*miaA*	Isopentenylpyrophosphate transferase
	K38	-, -	*prC*	C-terminal protease for penicillin binding protein
	K47	-, -	*atpH*	Membrane-bound ATP synthase
	K48	+, +	*ubiB*	NAD(P)H-flavin reductase
	K49	-, -	*ydiF*	Putative acetyl-CoA: acetoacetyl-CoA transferase
	K53	+, +	*tdK*	Thymidine kinase
	K65	+, +	*pps*	Phosphoenolpyruvate synthase
	K57	+, +	*ybjX*	Putative enzyme with unknown function
	K67	+, +	*pncA*	Nicotinamidase/pyrazinamidase
	K68	+, +	*rfaB*	Lipopolysaccharide 1,6-galactosyltransferase
	K71	+, +	*xapB*	Xanthosine permease
	K64	+, +	STM1940	Putative cell wall-associated hydrolase
	K74	+, +	*cbiA*	Cobyrinic acid a, c-diamide synthase
	K42	-, -	*aroA*	3-enolpyruvylshikimate-5-phosphate synthetase

a: The gene that was disrupted by the transposon insertion is listed. The annotation of *S*. Typhimurium LT2 genome is used if the transposon insertion was present in a gene which is uncharacterized and unnamed.

### Classification of the mutants according to the functions or putative functions of genes disrupted by the transposon insertions

The 54 mutants listed in Table 1 were classified according to the functions or putative functions of the ORFs disrupted by the transposons. We classified the mutants into the 12 categories: fimbrial biosynthesis and regulation, putative cytoplasmic protein, global regulator protein, cell membrane associated protein, cell envelope associated protein, periplasmic protein, ribosome modulation factor, type III secretion system protein, prophage-derived protein, sensor protein, transcription termination factor, and enzymes. Nine mutants that had defects in *fimA*, *fimC*, *fimD*, *fimH*, *fimY*, *fimZ*, *lpfB*, *pefI*, and *stbC *were placed in the fimbrial biosynthesis and regulation category. FimA is the major subunit of the type 1 fimbrial shaft, while the adhesion protein, FimH, confers the binding specificity of type 1 fimbriae [18,21]. FimC and FimD serve as the chaperone and molecular usher for type 1 fimbriae [18]. FimZ and FimY are positive regulators of *fimA *[14,19]. Since all these genes are involved in type 1 fimbrial biosynthesis and regulation, disruption of these genes caused the mutants to produce no fimbriae at all in either culture condition. Three mutants produced type 1 fimbriae in both culture conditions and had transposons inserted in other fimbrial related genes. K70 had a defect in *lpfB*, the chaperone protein for long polar fimbriae [22]. K61 had a defect in *pefI*, which involves the regulation of plasmid-encoded fimbriae [23]. K23 had a transposon inserted in *stbC *that encodes an outer membrane usher protein for Stb fimbriae in *S*. Typhimurium. The usher protein facilitates anchoring the developing fimbrial structure to the outer membrane [24].

The K1 and K28 mutants exhibited type 1 fimbriae in both static broth and on solid agar media. The gene products involved are putative cytoplasmic proteins, and their functions remain to be characterized. The K15 mutant had a transposon inserted in the *hupA *gene whose product is the histone-like DNA-binding protein, HU, a small, basic, thermostable protein involving DNA replication [25]. The K15 mutant and another mutant K46 that has disruption in *lrp *encoding leucine-responsive regulatory protein (LRP) did not exhibit type 1 fimbriae in either culture condition. Membrane associated proteins possess transmembrane domains. K17 had interruption of *yhjV *encoding protein similar to the tryptophan/tyrosine permease family in *Escherichia coli *[26]. The putative protein affected in K39 is a sodium/glucose cotransporter protein which belongs to a superfamily of membrane proteins responsible for the uphill transport of substrates coupled to the downhill transport of Na^+ ^[27]. The *mgtB *and *narK *genes, defective in K72 and K30 respectively, are involved in Mg^2+ ^and nitrate/nitrite transportation, respectively [28]. Disruption of the *rmf *gene impaired the 70S ribosome dimerization and resulted in inhibition of *S*. Typhimurium to produce type 1 fimbriae as seen in K56 [29]. The *ssaB*, *ssaV*, and *invB *belong to members of the type III secretion system, which is important in the pathogenesis of *S*. Typhimurium. The *ssaB *and *ssaV *mutants did not produce type 1 fimbriae in either culture condition, whereas the *invB *mutant produced type 1 fimbriae in both culture conditions. Two mutants, K11 and K19, possessed insertions in genes involved in synthesis of putative phage tail proteins [30]. K33 had an insertion in the gene encoding for a type III translocated protein, SseI, carried by the prophage Gifsy-2 [31]. Two mutants, K51 and K58, had transposons inserted into genes which encode the sensor part of the two-component regulatory system. SsrA is associated with the type III secretion system for the pathogenicity island II [32], while YehU in *S*. Typhimurium remains to be characterized. NusA binds to the RNA polymerase core enzyme and functions as a transcription termination factor [33]. The K73 carrying transposon in *nusA *did not produce type 1 fimbriae in either culture condition.

Among the mutants, 21 of 54 mutants (almost 39%) were in the category of enzymes comprising the largest group of the mutants. The enzymes detected in these mutants could be further divided into endoribonuclease (K3), restriction-modification enzyme (K5), protease (K38), tRNA modification enzyme (K43), the enzyme for lipopolysaccharide synthesis (K68), and primarily the enzymes required for general metabolism.

### Complementation test

Table 2 summarizes the results of the complementation test for K3 *cafA*, K5 *res*, K43 *miaA*, K48 *ubiB *and K73 *nusA *mutant strains. The K48 *ubiB *mutant strain produced type 1 fimbriae constitutively regardless grown in static broth or on solid agar culture condition. Transforming the plasmid pUbiB that contained the coding sequence of *ubiB *conferred the K48*ubiB *mutant to exhibit the type 1 fimbrial phenotype as seen in *S*. Typhimurium LB5010. Transforming a plasmid possessing the coding sequence of *res *to K5 *res *mutant did repress the type 1 fimbriae to express on solid agar as the parental strain. However, the complemented strain did not produce type 1 fimbriae in static broth culture. The complemented strains K3 *cafA *(pCafA), K43 *miaA *(pMiaA) and K73 *nusA *(pNusA) remained unchanged as their respective mutant strains, which did not produce type 1 fimbriae in either culture condition.

**Table 2:** Phenotypic expression of type 1 fimbriae by the selected mutant and the complemented strains

Strain	Plasmid transformed	Phenotypic expression of type 1 fimbriae by bacteria grown on or in^a^	
		
		agar	broth
LB5010	none	-	+
K3 *cafA*	none	-	-
K3 *cafA*	pCafA	-	-
K5 *res*	none	+	+
K5 *res*	pRes	-	-
K43 *miaA*	none	-	-
K43 *miaA*	pMiaA	-	-
K48 *ubiB*	none	+	+
K48 *ubiB*	pUbiB	-	+
K73 *nusA*	none	-	-
K73 *nusA*	pNusA	-	-

a: phenotypic expression of type 1 fimbriae was determined by mannose-sensitive yeast agglutination test.

### Reverse transcription polymerase chain reaction (RT-PCR) analysis

**Figure 3:**
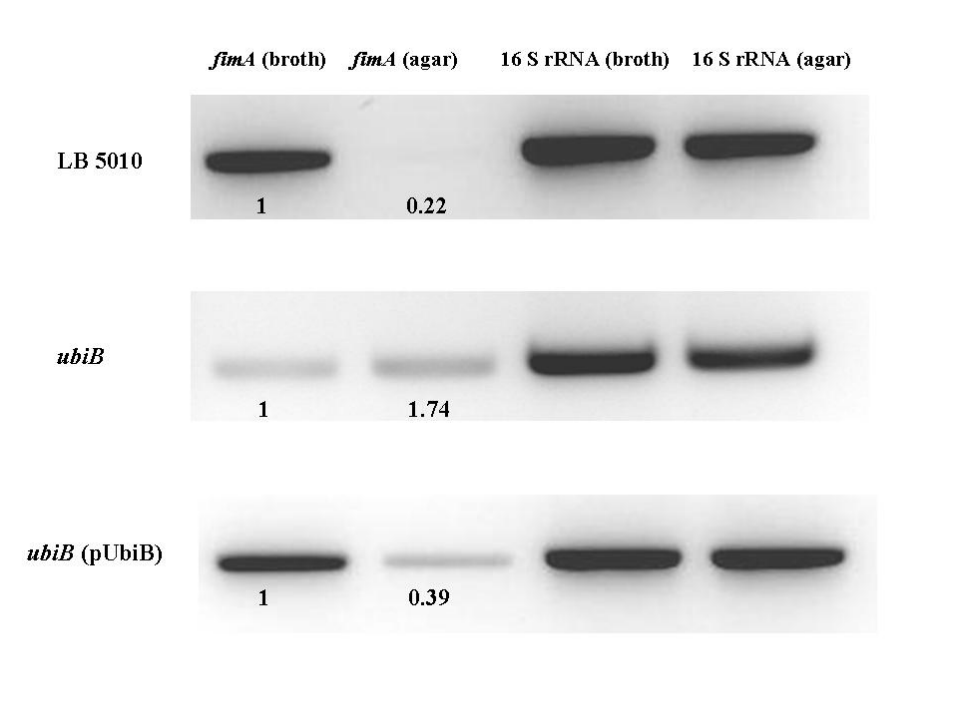
**RT-PCR for *fimA *transcription**. RT-PCR assays were used to monitor *fimA *and 16S rRNA transcription in the parental strain LB5010, *ubiB *mutant, and *ubiB *(pUbiB) strain. The intensities of the bands for each strain were determined by densitometry and expressed (Arabic numbers) relative to the value for *fimA *transcription obtained from the static LB broth culture condition. The intensities of 16S rRNA shown indicates that equivalent amounts of total RNA were used in the experiment.

Total RNA from the parental strain *S*. Typhimurium LB5010, K48 *ubiB *mutant, and K48 *ubiB *(pUbiB) were prepared and analyzed for *fimA *mRNA and 16S rRNA expression by RT-PCR. Figure 3 showed the RT-PCR analysis results. As for the parental strain LB5010, the *fimA *expression obtained from the static broth was approximately 4.5 fold (1: 0.22) of that obtained from the agar culture condition. For the K48 *ubiB *mutant, expression of *fimA *(static broth) was about 60% (1: 1.74) of the *fimA *(agar). When K48 *ubiB *strain harboured the plasmid pUbiB, *fimA *expression (broth) was about 2.6 fold (1: 0.39) of *fimA *(agar). As a control, 16S rRNA was constantly expressed in all the strains tested.

## Discussion

It is not surprising that many bacterial fimbriae have the ability to switch expression from a fimbriate to a non-fimbriate phenotype. Since bacterial population may face unpredictable milieu during their life cycle, having cells in both fimbriate and non-fimbriate phases offers the bacterial population an advantage of surviving in the diverse situations it will encounter [34]. The mechanisms that control the fimbrial regulation have been described in detail for only few fimbrial types. For example, the regulation in *E. coli *type 1 fimbriae is primarily controlled by inversion of a 314-bp segment of DNA (*fim *switch region) upstream of the *fimA *gene coding for the major fimbrial subunit [35]. Accessory proteins including the integration host factor (IHF), LRP and the histone-like nucleoid-structuring (H-NS) protein, also bind to the *fim *switch region to re-orientate the DNA into a configuration favourable for inversion [36-38]. Interestingly, the *E. coli *K-12 strain also possesses the *sfm *gene cluster (*sfmACDHFZU*), which is the orthologue of *S*. Typhimurium *fim *gene cluster [39]. Nevertheless not much characterization has been performed on *sfm*. The control system for type 1 fimbriae in *S*. Typhimurium differs from that of *E. coli*. No DNA inversion event has been detected and several *fim *gene products have been implicated as regulators of type 1 fimbrial expression [13-18,40]. Previous studies did not reveal that global regulator like IHF or H-NS play a role in *S*. Typhimurium type 1 fimbrial expression. In order to explore other elements outside the *fim *gene cluster that might also participate in type 1 fimbrial expression, we constructed a transposon library of *S*. Typhimurium. From the mutants that no longer exhibited the same type 1 fimbrial phenotypes as the parental strain whether grown on solid agar and in static broth cultures, 12 groups were classified according to the putative functions of the genes that were interrupted by the transposons. Not surprisingly, several mutants that had defects in the *fim *genes did not produce type1 fimbriae in either condition. The mutants possessing transposons in *fimA*, *fimC*, *fimD*, *fimH*, *fimZ*, and *fimY *all exhibited the predicted phenotypes. These *fim *genes are required for type 1 fimbrial biogenesis or regulation, and disruption of these genes causes the bacteria to be non-fimbriate in either condition. Interestingly, the present study identified 3 mutants that had defective genes for other fimbrial systems, and they produced type 1 fimbriae in both culture conditions. K23 had a transposon inserted in *stbC *that encodes the fimbrial usher protein for Stb fimbriae [41]. K70 had insertion in fimbrial chaperone protein for long polar fimbriae (Lpf). Those two gene products are involved in fimbrial biogenesis. How disruption of the outer membrane usher gene, *stbC*, of the Stb fimbrial system affects another fimbrial system poses an interesting research topic. We did try to complement the *stbC *mutant with a recombinant plasmid possessing the *stbC *coding sequence. However, the *stbC *mutant exhibited resistance to a battery of antibiotics, including ampicillin and chloramphenicol those frequently used in molecular cloning. This unexpected finding impeded us to transforming available plasmids into this strain for the time being. PefI identified in K61 is the homolog of PapI in *E. coli*. PefI negatively regulates the production of PefA, the major fimbrial subunit of plasmid-encoded fimbriae (Pef) [42]. Cross-talk between different fimbrial systems within the same microorganism has been demonstrated previously. For example, minor fimbrin of type 1 and F1C fimbriae of *E. coli *can be reciprocally exchanged to generate hybrid fimbriae with changed receptor specificities [43]. The regulator PapB of pyelonephritis-associated pili (Pap) prevents inversion of the *fim *switch controlling the expression of type 1 fimbriae in the same *E. coli *strain [44]. Recently Nuccio et al. also demonstrated that the presence of type 1 fimbrial biosynthesis genes interfered with the expression of plasmid-encoded fimbriae in *S*. Typhimurium [45]. Although additional research is required to elucidate the interaction of different fimbrial systems, our results did stress the observation that different types of fimbriae may coordinate for their own benefit.

A global regulator, LRP, has been demonstrated to play a role in the regulation of both type 1 fimbriae and P fimbriae of *E. coli *and Pef of *S*. Typhimurium. Recently McFarland et al. demonstrated that an *lrp *knockout *S*. Typhimurium strain did not produce type 1 fimbriae and Lrp is a positive regulator for *fim *gene expression by interacting with the *fimZ *promoter region [20]. FimZ is a positive regulator for *fimA *[14]. Their data suggested that a positive contribution of *fim *gene expression by Lrp/*fimZ *promoter interaction may be due to the displacement of H-NS, a repressor for *fimZ *[20,46]. The K46 mutant with the transposon inserted into *lrp *did not produce type 1 fimbriae in either culture condition. Our result concurred with their finding. The role that *lrp *plays in terms of type 1 fimbrial regulation in *S*. Typhimurium becomes apparent.

Several *S*. Typhimurium strains had transposons inserted in the genes that encode enzymes. The K3 strain had a defect in *cafA *that encodes a ribonuclease named RNase G in *E. coli*. Umitsuki et al. reported that RNase G was involved in the *in vivo *degradation of *adhE *mRNA which encodes a fermentative alcohol dehydrogenase, AdhE [47]. A mutation at *cafA *resulted in accumulation of *adhE *mRNA and prolonged the half-life of its mRNA. Interestingly, the K16 strain had a mutation in the gene that encodes alcohol dehydrogenase. The K16 strain constitutively expressed type 1 fimbriae. Overexpression of AdhE inhibited type 1 fimbrial expression, while the absence of AdhE caused type 1 fimbrial expression on solid agar or in static broth. How an enzyme involved in metabolism influences fimbrial expression is not clear, but previous studies demonstrated that type 1 fimbrial expression was linked to the nutrient status of the bacterial encounter. Broth culture supplemented with glucose favours *Shigella flexneri *remaining in a non-fimbriate state [6]. Morgenroth and Duguid described a *S*. Typhimurium biotype called the FIRN strain; it is non-type 1 fimbriate, non-inositol-fermenting, and non-rhamnose-fermenting [48]. The genes controlling fimbrial expression may be linked to the genes involved in carbohydrate metabolism. Interestingly, similar to the K16 strain, a variety of mutants in the present study having transposons inserted in the genes encoding "house-keeping" enzymes all constitutively exhibited type 1 fimbriae. Barak et al. used transposon mutagenesis to identify genetic determinants required for *S*. Newport to attach to plant tissue [49]. They also found that alcohol dehydrogenase, hydrolase, bacteriophage protein, and several membrane associated proteins, including transporter protein, similar to our findings, were the essential components for this *Salmonella *serotype to adhere to plant tissue [49]. The ability of *Salmonella *to colonize plant tissue and modulate type 1 fimbrial expression may share a common regulatory network.

A complementation test allowed us to examine if providing the coding sequence of the gene carried in a plasmid back to the mutant stain defective in such a gene would switch the phenotype of type 1 fimbrial expression to that originally observed. A comprehensive complementation test is not within the scope of the present study; however, we did test some strains. The complemented K3 *cafA *(pCafA), K43 *miaA *(pMiaA), and K73 *nusA *(pNusA) strains did not exhibit the type 1 fimbrial phenotype as the parental strain. Polar effect caused by the transposon could be one of the reasons. Another possibility could be due to the gene dosage effect conferred by the cloning vector. The K5 *res *(pRes) strain did repress the type 1 fimbrial production on solid agar as the parental strain LB5010. However, in static broth culture, the complemented strain did not exhibit type 1 fimbriae. The *res *gene, encoding for the restriction subunit of type III restriction-modification (R-M) system of *S*. Typhimurium, locates upstream of the *mod *gene which encodes the methyltransferase subunit of R-M system. Since Res subunit requires Mod to form a complex to recognize and cleave DNA [50], failure of *mod *expression caused by polar effect could account for the partially complemented phenotype of the K5*res *(pRes) strain. NusA is a transcription termination factor and under some circumstance it may also function as a transcription elongation factor [51]. The ability of NusA to enhance RNA polymerase pausing would result in tight coupling of transcription and translation, thus interferes with the Rho-mRNA interactions and consequently blocks termination of transcription [52]. The transposon inserted in *nusA *in K73 strain could cause transcription/translation coupling defect in a variety of genes including those that may affect *fim *expression. The pNusA transformed in K73 strain may not produce precise stoichiometric ration of NusA, leading K73 *nusA *(pNusA) strain remained unchanged as the K73 *nusA *strain. MiaA is an isopentenylpyrophosphate transferase that is required for tRNA modification. Ericson and Björk demonstrated that a *miaA *mutant in *S*. Typhimurium LT2 which was deficient in 2-methylthio-*N*6-(4-hydroxyisopentenyl) adenosine (ms^2^io^6^A37) in its tRNA induced pleiotropic effects on bacterial physiology such as reduced growth rate and polypeptide chain elongation rate [53]. Non-fimbriate phenotype exhibited by K43 *miaA *strain in both culture conditions could be the result of such pleiotropic effects. The K48 *ubiB *mutant transformed with the pUbiB plasmid restored the type 1 fimbrial phenotype as the parental strain LB5010. The K48 *ubiB *(pUbiB) cultured on agar did not agglutinate yeast cells. Although signals of both LB5010 and K48 *ubiB *(pUbiB) from agar could be detected by densitometry in RT-PCR assay, we argued that such *fimA *amount would not be enough for scoring positive in yeast agglutination test. Our complementation assay did prove that the *ubiB *mutant could exhibit the same phenotype as LB5010 when it harboured a plasmid containing the coding sequence of *ubiB*. The *ubiB *in *S*. Typhimurium could encode a NAD(P)H-flavin-reductase, whose homologue in *E. coli *has been proved to be required for the first monooxygenase step in ubiquinone biosynthesis [54]. How a gene involving in biosynthesis of ubiquinone, an essential component of electron transport chain influences type 1 fimbrial expression is currently unknown. However, Leonard et al. reported that UbiB protein contains motifs found in eukaryotic-type protein kinase [55]. It was speculated that phosphorylation may be required for ubiquinone biosynthesis-activating proteins [55]. Possibly UbiB would also phosphorylate another substrate protein that affects *fimA *expression. The function of *ubiB *in *S*. Typhimurium and the relationship of *ubiB *and *fim *expression demands more careful characterization. Our data clearly showed that an *ubiB *mutant produced *fimA *at the condition that either favours or inhibits type 1 fimbrial expression and complementing this mutant with a plasmid possessing *ubiB *coding sequence restored it to the phenotype similar to the parental strain.

The present study revealed that the genetic determinants other than the *fim *genes could have the potential to affect type 1 fimbrial expression in response to different standard laboratory culture conditions. However, a battery of genes identified by transposon mutagenesis still requires detail characterization to validate its linkage to fimbrial expression. The phenotype caused by polar effect is one of the drawbacks in this kind of study. Validation of the genes of interest that are associated with type 1 fimbrial expression in *S*. Typhimurium is presently under investigation in our laboratory.

## Conclusion

The ability to switch between fimbrial expressions in bacteria may be beneficial for survival. Fimbriate bacteria may facilitate colonizing host cells at certain stages of infection, while non-fimbriate bacteria may also have their own role to play, for example, as in avoiding recognition by phagocytes, or allowing the bacteria to shed and colonize another site on the host. Compared to *E. coli*, *Salmonella *has a higher survival rate in the external environment, which promotes transmission to a new host [56]. Persistent infection of *Salmonella *in the environment is enhanced by adhesion and biofilm formation, both of which involve fimbrial appendages [21]. How *S*. Typhimurium fluctuates between type 1 fimbriate phase and non-fimbriate phase *in vivo *remains to be uncovered. Nonetheless the present study reveals that several genetic determinants may be associated with the ability of *S*. Typhimurium to modulate type 1 fimbrial expression between solid agar and static broth culture conditions. These two laboratory culture conditions may to some extent mimic the environmental milieu that *Salmonella *would encounter. We report here that other gene products besides those in the *fim *gene cluster are also required for type 1 fimbrial expression. How each gene influences type 1 fimbrial expression is an interesting research topic which warrants further investigation.

## Methods

### Bacterial strains, media, culture conditions, primers and plasmids

The *S*. Typhimurium strain used in the present study is *S*. Typhimurium LB5010, a LT2 strain derivative [57]. This strain produces type 1 fimbriae and is fimbrial phase variable [57]. *E*.*coli *JM109 strain was used for molecular cloning [58]. The primers and plasmids used in the present study are listed in Table 3 and Table 4, respectively. Bacteria were grown in Luria-Bertani (LB) broth (Difco/Becton Dickinson, Franklin Lakes, NJ) or plated on LB agar [59]. Media were supplemented with antibiotics when required at the following concentrations: kanamycin, 50 μg/ml; ampicillin, 100 μg/ml; chloramphenicol, 20 μg/ml. The above antibiotics were obtained from Sigma (St. Louis, MO).

**Table 3:** Oligonucleotide primers used in the present study

Primer	Sequence (5'-3')
kan-5	TAACATCATTGGCAACGCTACCT
kan-6	GCATCGGGCTTCCCATACAATCG
kan-7	GTCGCACCTGATTGCCCGACATT
cafA-F	ATACGACTCACAACCTTGCTTTGCCGGACG
cafA-R	TTTCTGCGCAGGATATTAGTGGCTATGTCG
res-F	CATTGTCATTTACGGCTACTCT
res-R	ACGATAACCTTCAAGTCAAC
miaA-F	CTGCTGGCGGATGTTGAGCGGCTATGT
miaA-R	CGCAATGCGTTCAGGAACGGATCTTGT
nusA-F	CCGTCCTATGTTCACTGCCGAT
nusA-R	CTGCTGTACCAGGCGATCCACGGAAAC
ubiB-F	GGTCGTCCTATTGTTAAAGATCCTGA
ubiB-R	CGCAATACAAAGCCTGTAGATATTCA
16S-F	TTCCTCCAGATCTCTCTACGCA
16S-R	GTGGCTAATACCGCATAACG
fimA-F	ACTATTGCGAGTCTGATGTTTG
fimA-R	CGTATTTCATGATAAAGGTGGC

**Table 4:** The plasmids used in the present study

Plasmid	Genotype or relevant features^a^	Reference or source
pUC4K	3.9 kb vector, Kan^r ^Am^r^	Amersham Biosciences
yT&A	2.7 kb cloning vector; Am^r^	Yeasten Biotech
pCafA	1.3 kb DNA fragment containing *cafA *cloned into yT&A; Am^r^	This study
pRes	3.7 kb DNA fragment containing *res *cloned into yT&A; Am^r^	This study
pMiaA	1.2 kb DNA fragment containing *miaA *cloned into yT&A; Am^r^	This study
pNusA	1.8 kb DNA fragment containing *nusA *cloned into yT&A; Am^r^	This study
pUbiB	988 bp DNA fragment containing *ubiB *cloned into yT&A; Am^r^	This study

a: Kan^r^, kanamycin resistant; Am^r^, ampicillin resistant.

### Construction of a transposon mutant library of *S*. Typhimurium

A transposon mutant library of *S*. Typhimurium was constructed using the EZ::TN <KAN-2> Tnp Transposome system (Epicentre, Madison, WI). This system contains the transposon Tn903 which possesses a kanamycin resistance cassette. Briefly, 1 μl of the transposome mixture was placed in 40 μl of *S*. Typhimurium-competent cells and electroporated into the cells for transposition to occur. The electroporation conditions were set as follows using ECM 630 (BTX, Pittsburgh, PA): voltage of 1.70 kV, resistance of 125 ohms, and capacitance of 50 μF. Immediately after electroporation, 1 ml of S.O.C medium (Invitrogen, Carlsbad, CA) was added to the cuvette and the medium/cell mixture was transferred to a tube and incubated at 37°C with constant shaking for 1 h. Four milliliters of LB broth was added to dilute the bacterial cells, and a-100 μl of aliquot was evenly spread on the kanamycin-containing LB agar.

### Screening of *S*. Typhimurium transposon mutants that exhibited different type 1 fimbrial phenotypes than the parental strain

The transformants grown on LB agar supplemented with kanamycin were randomly selected. Each colony was streaked on LB agar and then inoculated into 10 ml of LB broth. The LB agar plates were incubated at 37°C for 18 h while the broth preparations were incubated statically at 37°C for 48 h. Bacterial cells from the solid agar were collected by a sterile loop and resuspended in 100 μl of 1 × phosphate-buffered saline (PBS). Cells in the broth medium were collected by centrifugation, and the pellet was resuspended in 100 μl of 1 × PBS. Subsequently, 30 μl of a 3% (vol/vol) suspension of *C*.*albicans *in PBS and an equal amount of bacterial cells to be tested were mixed together on a glass slide [11]. Visible agglutination after gentle agitation indicated a positive reaction for the presence of type 1 fimbriae. Any bacterial suspension that produced type 1 fimbriae was further mixed with *C. albicans *along with 3% (wt/vol) of a D-mannose solution (Sigma). The mannose-sensitive agglutination conferred by type 1 fimbriae was inhibited in the presence of mannose.

### Southern hybridization

Southern hybridization analysis was used to detect the presence of the kanamycin resistance cassette on the *S*. Typhimurium genomic DNA. Genomic DNAs from *S*. Typhimurium LB5010 and the mutant strains that were no longer type 1 fimbrial phase variable were isolated using a MasterPure DNA Purification Kit (Epicentre). The genomic DNA preparations were cleaved by *Eco*RI, and the DNA fragments were separated by electrophoresis in a 0.7% agarose gel. *Eco*RI was chosen to cut the genomic DNA since there was no *Eco*RI site within the kanamycin resistance cassette of Tn903. A DNA probe derived from the kanamycin resistance cassette sequence was hybridized to one band in the Southern blot if the transposition had occurred only once. The DNA was soaked in a denaturing solution (0.5 M NaOH and 1 M NaCl) twice for 15 min each time and a neutralization solution (3 M NaCl, 0.5 M Tris-HCl) for 30 min with continuous agitation at room temperature. The DNA was then transferred onto a nylon membrane by a vacuum blotter (trans-Vac, Hoefer, San Francisco, CA) with 10 × SSC and cross-linked to the membrane by exposure to short-wavelength UV light from a UV Spectrolinker (Spectronics, Westbury, NY). The 1.3-kb kanamycin resistance cassette-containing DNA fragment was cleaved by *Hind *III from the pUC4K plasmid and isolated from the agarose gel using a Montage Gel Extraction Kit (Millipore, Bedford, MA). Probe labeling and hybridization were performed using a DIG High Prime DNA Labeling and Detection Starter Kit II (Roche Diagnostics, Mannheim, Germany) according to the protocols provided by the manufacturer. The 1.3-kb DNA probe was labeled by the randomly primed incorporation of digoxigenin-labeled dUTP. Briefly, hybridization to the blot was performed at 68°C in a standard buffer (5 × SSC, 0.1% N-lauroylsarcosine, 0.02% SDS, and 1% blocking reagent) for 16 h. Two 5-min washes were performed at room temperature in a 2 × SSC (1 × SSC is comprised of 0.15 M NaCl and 0.015 M sodium citrate)-0.1% SDS solution. The blot was then washed with two 15-min washes in 0.1 × SSC-0.1% SDS solutions at 68°C. Hybrids were detected using an antibody conjugate (anti-digoxigenin-alkaline phosphate conjugate) and NBT/BCIP (18.75 mg/ml nitrobluetetrazolium chloride and 9.4 mg/ml of a 5-bromo-4-chloro-3-indolyl-phosphate in 67% [vol/vol] DMSO) solution.

### Identification of transposon insertion sites

The transposon insertion sites in the mutants of interest were identified by a three-step PCR using a DNA Walking SpeedUp Premix Kit (Seegene, Seoul, Korea). This kit uses annealing control primer (ACP) technology [60,61]. The DW-ACP primers provided by the kit are designed to capture unknown target sites, and with primers internal to the known sequence, will amplify DNA fragments possessing the junction region of known and adjacent sequences. The first PCR reaction was performed independently in four individual PCR tubes using the kan-5 primer internal to the kanamycin resistance cassette with one of the DW-ACP 1, 2, 3, or 4 primers. The total 50 μl of the PCR contained genomic DNA of the mutant under study, 4 μl of the DW-ACP primer, the kan-5 primer, distilled water, and 25 μl of 2 × SeeAmp ACP Master Mix II. The PCR program was set as follows: one cycle at 94°C for 5 min, 42°C for 1 min, and 72°C for 2 min; followed by 30 cycles of 94°C 30 s, 55°C 30 s, and 72°C for 100 s. Finally the PCR was extended at 72°C for 7 min. The product from the first PCR was purified using Montage PCR Filter Units (Millipore) and was used as the template for the second PCR. The second PCR with a final volume of 20 μl contained the first PCR product as the template, 1 μl DW-ACPN, a kanamycin resistance cassette-specific kan-6 primer upstream from kan-5, distilled water, and 10 μl of 2 × SeeAmp ACP Master MixII. The PCR program was set as follows: denaturation at 94°C for 3 min; 35 cycles of 94°C 30 s, 56°C 30 s, and 72°C 100 s; then the PCR was extended at 72°C for 7 min. The third PCR used 2 μl of the second PCR product (without purification) as the template. The remaining components consisted of 1 μl universal primer, 1 μl of the kan-7 primer upstream from kan-6, distilled water, and 10 μl of 2 × SeeAmp ACP Master MixII. The third PCR reaction consisted of 94°C for 3 min; followed by 35 cycles of 94°C for 30 s, 60°C for 30 s, and 72°C for 100 s; then the PCR was extended at 72°C for 7 min. The products from the third PCR were purified from the agarose gel and sequenced by using the kan-7 primer to determine the junction sequence where the transposon was inserted into the genomic DNA. The sequence of the junction region of the transposon was compared against the *S*. Typhimurium LT2 genome sequence [GenBank: NC 003197].

### Complementation Test

Primers used for complementation test (cafA-F, cafA-R, res-F, res-R, miaA-F, miaA-R, nusA-F, nusA-R, ubiB-F and ubiB-R) were listed in Table 3 and were used to amplify genomic DNA of *S*. Typhimurium LB5010. The PCR product that contained the full coding sequence of the respective gene was cloned into the yT&A vector (Yeasten Biotech, Taipei, Taiwan) according to the protocol provided by the manufacturer. The resulting plasmid was transformed into the corresponding mutant strain by electroporation. The complemented strains were tested for type 1 fimbrial expression in static broth and on solid agar medium by yeast agglutination test.

### Reverse transcription polymerase chain reaction (RT-PCR) analysis

The bacteria were stabilized by adding RNAprotect bacteria reagent (Qiagen, Valencia, CA). Total RNA was prepared by using an RNeasy Mini Kit (Qiagen) and RNase-free DNase (1 unit/1 μg RNA) according to the manufacturer's protocol. RT-PCR was performed using a Fast-Run HotStart RT-PCR (AMV) kit (Protech, Taipei, Taiwan). Briefly, RNA was denatured at 58°C for 5 min, followed by cDNA synthesis at 42°C for 30 min and inactivation at 94°C for 2 min. The following PCR conditions are: 35 cycles of denaturation at 94°C for 30 s, annealing at 54°C for 30 s, and extension at 72°C for 30 s. An additional extension was performed at 72°C for 5 min. Primers fimA-F and fimA-R were used to detect the mRNA expression of *fimA*, while 16S-F and 16S-R primers were used to detect the mRNA expression of 16S rRNA as a control. Densitometry was performed using the Image Quant version 5.2 image analysis software (Molecular Dynamics, GE Healthcare, UK).

## Authors' contributions

Y-CC drafted the manuscript. K-CW participated in the sequence alignment and molecular cloning experiments. Y-TC, C-HY, S-CM, and C-CF were responsible for the library screening, molecular cloning, and hybridization experiments. L-HC carried out the complementation tests. K-SY conceived and coordinated the study, and helped to draft the manuscript. All authors read and approved the final manuscript.
